# Mitral valve replacement using a collar-reinforced mitral prosthesis for severe mitral annular calcification, which secures implantation in both the supra-annular and intra-atrial positions: a case report

**DOI:** 10.1186/s13019-023-02429-5

**Published:** 2023-11-11

**Authors:** Atsushi Morishita, Hideyuki Tomioka, Seiichiro Katahira, Takeshi Hoshino, Kazuhiko Hanzawa

**Affiliations:** 1 Department of Cardiovascular Surgery, Numata Neurosurgery Heart-Disease Hospital, 8 Sakae-cho, Numata, 378-0014 Japan; 2 Department of Cardiovascular Surgery, Saitama Eastern Cardiovascular Hospital, Koshigaya, Japan; 3 Division of Health Administration, Hamakawasaki Operation Center, Toshiba Human Asset Service Corporation, Kawasaki, Japan; 4 Department of Anesthesiology, Minami Machida Hospital, Machida, Japan; 5 Department of Advanced Treatment and Prevention for Vascular Disease and Embolism, Niigata University Graduate School of Medical and Dental Sciences, Niigata, Japan

**Keywords:** Mitral annular calcification, Mitral valve replacement, Mitral prosthesis, Supra-annular and intra-atrial position

## Abstract

**Background:**

Appropriate management of mitral annular calcification associated with mitral valve surgery must be determined on a case-by-case basis. However, an established procedure remains uncertain.

**Case presentation:**

This report describes a surgical case of severe mitral and aortic valve stenosis associated with severe mitral annular calcification in a 71-year-old woman who underwent mitral valve replacement with a collar-reinforced mitral prosthesis. The patient underwent surgical repair after the treatment for heart failure. As the present patient was deemed to be at high risk for conventional mitral valve replacement, we applied a composite prosthetic valve that was enlarged circumferentially on the ventricular side of the prosthesis with a bovine pericardial patch. First, the solid calcium bar was left untouched and only the friable calcified mass that was easily scattered was removed. Subsequently, the prosthesis was secured by two mattress sutures placed in the intra-atrial position at the region of the extended calcified myocardium. Additionally, ten mattress sutures were placed in the supra-annular position at the other regions capable of passing stitches from the ventricular side to the atrial side. Finally, a 1.5 cm wide trimmed bovine pericardial collar was sutured circumferentially from the annulus to the atrial wall using running 4–0 polypropylene for reinforcement. Although temporary hemodialysis was performed for acute renal failure, the patient remained asymptomatic.

**Conclusions:**

The present case suggests that mitral valve replacement using a collar-reinforced mitral prosthesis may be an effective technique for severe mitral annular calcification. To avoid catastrophic complications associated with treatment for severely calcified annulus, it is crucial to make a prudent preoperative decision regarding the surgical strategy under circumstances where conventional mitral valve replacement is impossible.

## Background

Frequent severe mitral annular calcification (MAC) necessitates complicated techniques different from conventional mitral valve replacement (MVR) [1–7]. Extensive calcium debridement may cause atrioventricular disruption and result in hematoma, left circumflex coronary artery injury, stroke, embolization, and left ventricular posterior wall rupture [8]. When the placement of valve sutures in an intra-atrial position in the severely calcified annulus cannot be avoided, a bovine pericardial collar as an adjunct is considered useful to prevent complications such as periprosthetic leakage, hemorrhage, and subsequent aneurysm formation. Herein, we describe the novel case of a patient who underwent double valve replacement that included MVR with a collar-reinforced mitral prosthesis while avoiding manipulation for severe MAC to the possible extent.

## Case presentation

**Fig. 1:**
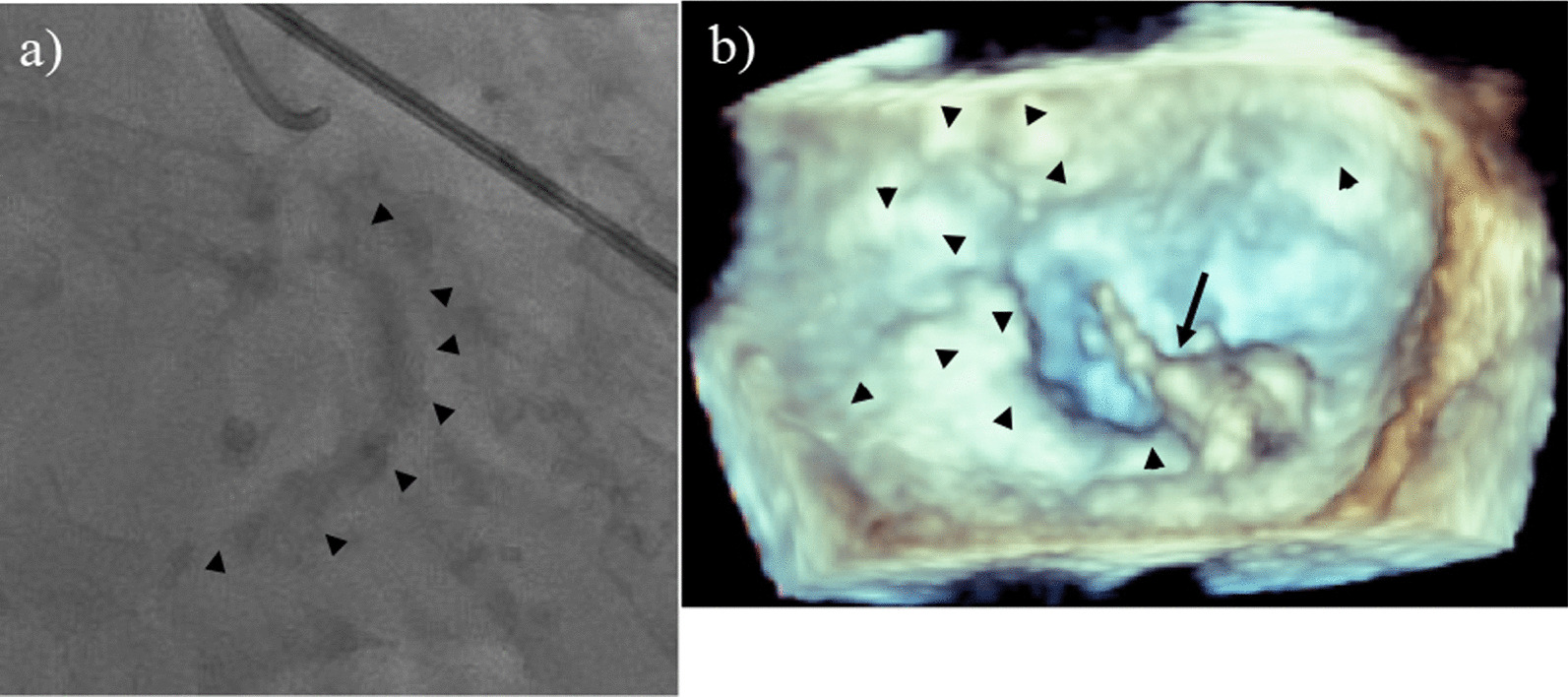
Preoperative cardiac fluoroscopy and 3-dimensional transesophageal echocardiography. **a** Preoperative cardiac fluoroscopy shows calcification (black arrowheads) around the posterior mitral annulus. **b** Preoperative 3-dimensional transesophageal echocardiography demonstrates extensive calcification of the mitral anterior and posterior annuli (black arrowheads) and prolapse and torn chordae of the posteromedial scallop of the posterior leaflet (black arrow)

A 71-year-old woman was admitted to our hospital by ambulance with New York Heart Association functional class IV. An artificial respirator was placed immediately after arrival with complaints of orthopnea. The patient had been treated for hypertension, hyperuricemia, dyslipidemia, diabetes, and chronic kidney disease. The pulse rate of the patient was regular at 110 beats/min and blood pressure was 130/60 mmHg. Her height, weight, and body surface area were 153 cm, 71.3 kg, and 1.69 m^2^, respectively. Chest radiography revealed moderate cardiomegaly with a cardiothoracic ratio of 56.1% and severe pulmonary venous congestion. Electrocardiography disclosed sinus tachycardia. On cardiac auscultation, grade IV/VI systolic and diastolic murmurs were observed at the left sternal border and apex. Transthoracic echocardiography showed that the mean pressure gradients of the mitral and aortic valves were 15 mmHg and 74 mmHg, respectively. This was associated with severe mitral and tricuspid regurgitation and a good left ventricular ejection fraction of 64%. Coronary angiography revealed no significant stenosis, however, cardiac fluoroscopy showed calcification around the posterior mitral annulus (Fig. 1a). Transesophageal echocardiography (TEE) demonstrated severe mitral valve stenosis including extensive calcification of the mitral posterior annulus (Fig. 1b), which extended into the myocardium of the left ventricle partially. Severe aortic valve stenosis included three severely calcified aortic cusps and fused commissures. Holter electrocardiography revealed paroxysmal atrial fibrillation. After treatment for heart failure with the support of intra-aortic balloon pumping, we decided to perform surgical treatment for both mitral and aortic valve stenosis after obtaining written informed consent.

**Fig. 2:**
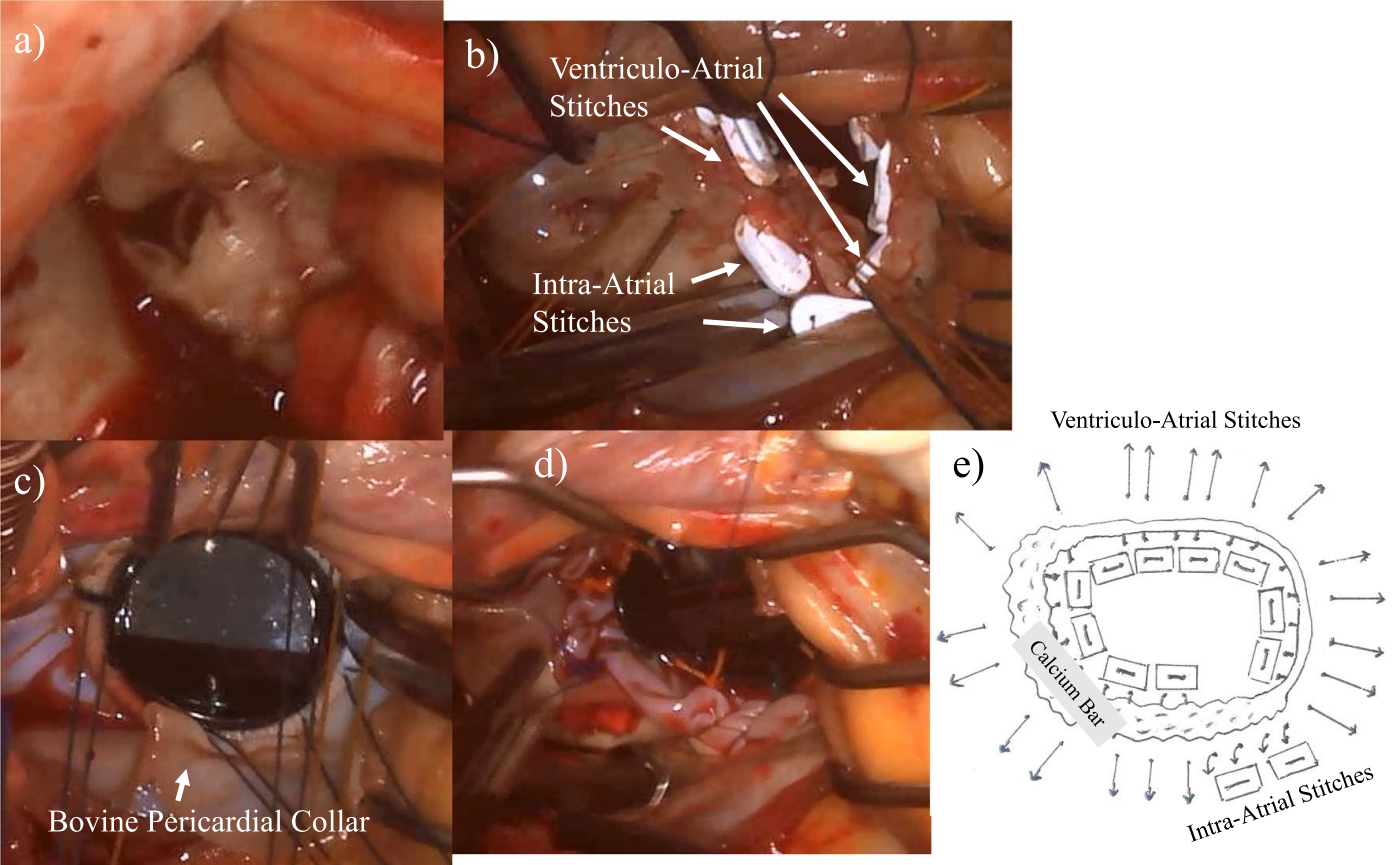
Intraoperative photographs and schema. **a** Intraoperative photographs demonstrates severe mitral stenosis, **b** two intra-atrial stitches and other ventriculo-atrial stitches, **c** the seating of the composite valve, **d** reinforcement by the trimmed bovine pericardial collar, and **e** schema of the valve suture placement showing the two intra-atrial mattress sutures and ten ventriculo-atrial mattress sutures

**Fig. 3:**
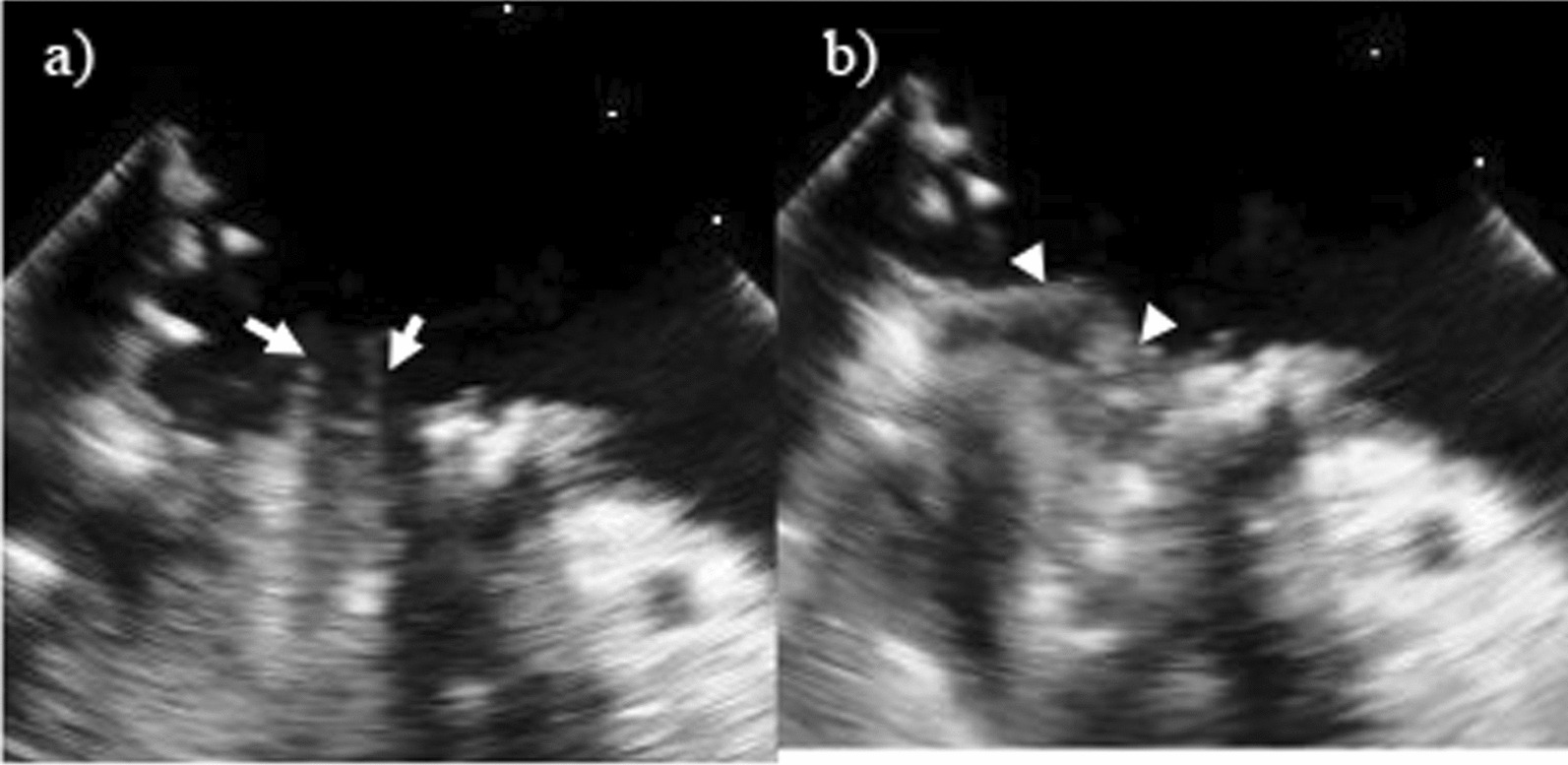
Postoperative transesophageal echocardiography. Postoperative transesophageal echocardiography reveals that the mitral prosthetic valve is functioning normally at both the **a** open (white arrows) and **b** closed (white arrowheads) positions, without perivalvular leakage

Aortotomy was performed after establishing standard cardiopulmonary bypass and cardioplegic arrest. Myocardial protection was provided by using an intermittent antegrade and retrograde cold blood cardioplegia and topical cooling. Subsequently, heavily calcified aortic valves were excised, and the remaining annular calcified mass was decalcified and carefully removed piece by piece using a rongeur. A 16-mm ATS AP360 valve (ATS Medical Inc., Minneapolis, MN, USA) was placed in the supra-annular position with non-everting mattress sutures. Through longitudinal left atriotomy, a calcium bar with a thickness of more than 1 cm was placed along the posterior mitral annulus from the anterolateral commissure to the posteromedial scallop of the posterior leaflet. Calcification extended into the myocardium of the left ventricle at a portion of the posteromedial scallop of the posterior leaflet, where prolapse and torn chordae were observed (Fig. 2a). The motion of the anterior mitral leaflet was severely restricted. A calcified protrusion was observed on the ventricular side via the posteromedial commissure of the mitral anterior annulus. Overall, calcification occurred in more than half of the circumferential annuli. Finally, the anterior mitral leaflet and posteromedial scallop of the posterior mitral leaflet were excised. Prudent care was taken not to leave fragile calcium fragments that were highly likely to scatter. The calcium bar involving two-thirds of the posterior annulus was hard on palpation and remained intact because extensive debridement was considered hazardous. At the portion in which calcification was extended into the myocardium, two 2–0 polyester mattress pledgeted sutures were placed in the left atrial wall approximately 1 cm lateral to the mitral posterior annulus. In other regions, stitches were passed from the ventricular side to the atrial side using the remaining ten mattress sutures (Fig. 2b). Particularly in the calcium bar, careful valve suture placement was performed while deliberately investigating the fibrous area with the needle tip through the calcium deposits and the surrounding fibrous tissue. All sutures were interrupted to place the outlets of every stitches on the same plane. Initially, the pre-assembled composite valve, which was composed of a 25-mm St.Jude valve (St. Jude Medical Inc., St. Paul, MN), enlarged the circumference of the sewing ring by attaching a bovine pericardial collar. It was secured to the mitral annulus in a manner allowing the prosthetic valve opening and the calcium bar met to meet at right angles to each other. The prosthetic leaflets did not interfere with the residual calcified valvular tissue (Fig. 2c). Subsequently, a 1.5 cm wide trimmed bovine pericardial collar was sutured around the annulus to the atrial wall using running 4–0 polypropylene for reinforcement (Fig. 3d). Additionally, concomitant procedures, which consisted of the De Vega method, were contracted to 29 mm for severe tricuspid regurgitation and pulmonary vein isolation using bipolar radiofrequency ablation for paroxysmal atrial fibrillation. The weaning from cardiopulmonary bypass was successful and the mean bypass and cross-clamp times were 272 min and 212 min, respectively. The intra-aortic balloon pumping was removed on postoperative day 5. Postoperative TEE revealed normal functioning of the mitral prosthetic valve, without perivalvular leakage (Fig. 3). Although temporary hemodialysis was performed for acute renal failure, the patient was discharged on postoperative day 57 without another complications. She was followed-up once a month after discharge from the hospital. Anticoagulation therapy with warfarin achieved a prothrombin time- international normalized ratio of 2.0–3.0. Oral medications with bisoprolol fumarate (2.5 mg/day) and sacubitril valsartan sodium hydrate (200 mg/day) were administered. The 1-year transthoracic echocardiography after surgery showed a mean pressure gradient of the mitral prosthetic valve of 5 mmHg without perivalvular leakage.

## Discussion and conclusions

The appropriate procedure for mitral annular calcification in mitral valve surgery must be selected on a case-by-case basis concerning the range and properties of the calcification [8]. The approach for the calcium bar involves avoiding manipulation of the calcified mass, partial decalcification, and complete decalcification following annular reconstruction. Although extensive calcium debridement enable implantation of a larger prosthesis, it may cause atrioventricular disruption and result in hematoma, left circumflex coronary artery injury, stroke, embolization, and left ventricle posterior wall rupture. Feindel et al. and Mihaljevic et al. favor intra-annular insertion of a mitral prosthesis after the removal of the calcium bar and the creation of a new annulus for MAC [1, 2]. A previous study demonstrated that the operative mortality of mitral valve surgery with MAC was higher (9.3%) than that without MAC (1.5%) during the same period, even with a complicated technique. A study reported an in-hospital mortality rate of 20% with no relevance to the operative technique. With the widespread use of therapies for structural heart disease, transcatheter MVR has been initiated as the treatment of choice for severe MAC in patients with prohibitive surgical risk. However, it is uncertain whether long-term results can be evaluated [9]. Furthermore, this procedure has not yet been approved for use in Japan. Another approach involves the intra-atrial insertion of a mitral prosthesis, which enlarges the circumference of the sewing ring with a Dacron collar, as described by Nataf et al. and Sakamoto et al. [3, 4]. However, we were concerned about periprosthetic leakage, hemorrhage of the fragile left atrial wall, and subsequent aneurysm formation. The technique of sealing between the calcified mitral annulus and the sewing ring of the prosthesis with the materials, such as equine pericardial patch, PTFE felt, and transferred anterior mitral leaflet, may be valuable adjuncts, as reported by Okita et al., Hussain et al., and Nezic et al. [5–7]; both intra and supra-annular insertion of a mitral prosthesis are acceptable. To prevent the occurrence of complications, we decided to remove only the friable calcified mass, which is easily scattered, and leave the solid calcium bar untouched. Furthermore, it was important to completely cover the area of the decalcified annulus to circumvent systemic embolization of calcium fragments. Additionally, providing a satisfactory visual field of annular reconstruction in the small left ventricular cavity is challenging. Finally, we needed to avoid prolonging the aortic cross-clamp time in a failing heart. Hence, we sutured the prosthetic valve to the atrial wall in a region where sutures could not pass through the calcified annulus. This may have resulted in periprosthetic leakage and hemolytic anemia due to the dehiscence of the prosthetic valve. In the other region, non-everting mattress sutures could be placed in the annulus with appropriate strength without placing a calcium bar into the disorder. As the present patient was deemed to be at high risk for conventional MVR, we applied a composite prosthetic valve that was enlarged circumferentially on the ventricular side of the prosthesis with a bovine pericardial patch. The bioprosthetic valve was not selected, fearing that the stent strut would cause left ventricular rupture, and a smaller prosthesis size was required for implantation owing to double valve replacement. The bovine pericardial patch, which was flexibly held between the native mitral annulus and the sewing cuff of the prosthesis, was considered a suitable material for support and stability. Reinforcement of anchoring of the pericardial patch edge all over the the left atrial wall circumference plays an important role in distributing and evenly buffering the effect on the prosthesis with high left ventricular pressure and reducing the risk of periprosthetic leakage, even if two sutures were placed on the atrial wall. The chosen St. Jude Medical valve is advantageous owing to its low-profile design, minimizes leaflet exposure below the prosthesis, and appears to be feasible to avoid impingement of residual mitral leaflets and subvalvular apparatus. The anti-anatomical implantation of a mitral prosthesis reduces the risk of valve thrombosis and hemolysis [10]. However, this direction was not chosen because increasing the clearance below the mitral prosthesis was a higher priority. However, proper management using anticoagulation therapy is mandatory. Additionally, TEE is a useful modality for the accurate preoperative identification of calcium bars and postoperative assessment of implanted prostheses. TEE plays a crucial role in determining whether conventional MVR can be performed prior to surgery and the required treatment strategy. MVR with a collar-reinforced mitral prosthesis is as an effective alternative technique for patients with severe MAC. The present case highlights the importance of the MAC approach, which consists of removing only the friable calcified mass and leaving the solid calcium bar untouched, to alleviate catastrophic complications due to extensive calcium debridement. Additionally, it is useful for securing the seating of the prosthesis by a double-layer structure that was placed in both supra-annular and intra-atrial positions, while ensuring that the placement in the intra-atrial position is avoided whenever possible.

## Data Availability

The datasets supporting the conclusions of this article are included within the article.

## Abbreviations

MAC: Mitral annular calcification
MVR: Mitral valve replacement
TEE: Transesophageal echocardiography
